# The C-C Chemokine Receptor Type 4 Is an Immunomodulatory Target of Hydroxychloroquine

**DOI:** 10.3389/fphar.2020.01253

**Published:** 2020-08-28

**Authors:** Tyler C. Beck, Kyle R. Beck, Calvin B. Holloway, Richard A. Hemings, Thomas A. Dix, Russell A. Norris

**Affiliations:** ^1^ Dix Laboratory, Department of Drug Discovery & Biomedical Sciences, Medical University of South Carolina, Charleston, SC, United States; ^2^ Norris Laboratory, Department of Regenerative Medicine and Cell Biology, Medical University of South Carolina, Charleston, SC, United States; ^3^ College of Medicine, Medical University of South Carolina, Charleston, SC, United States; ^4^ College of Pharmacy, The Ohio State University, Columbus, OH, United States; ^5^ Pritzker School of Medicine, The University of Chicago, Chicago, IL, United States

**Keywords:** COVID-19, hydroxychloroquine, CCR4, SARS-CoV-2, immunomodulation

## Abstract

The emergence of a severe acute respiratory syndrome coronavirus 2 (SARS-CoV-2; COVID-19) in China, reported to the World Health Organization on December 31, 2019, has led to a large global pandemic and is a major public health issue. As a result, there are more than 200 clinical trials of COVID-19 treatments or vaccines that are either ongoing or recruiting patients. One potential therapy that has garnered international attention is hydroxychloroquine; a potent immunomodulatory agent FDA-approved for the treatment of numerous inflammatory and autoimmune conditions, including malaria, lupus, and rheumatoid arthritis. Hydroxychloroquine has demonstrated promise *in vitro* and is currently under investigation in clinical trials for the treatment of COVID-19. Despite an abundance of empirical data, the mechanism(s) involved in the immunomodulatory activity of hydroxychloroquine have not been characterized. Using the unbiased chemical similarity ensemble approach (SEA), we identified C-C chemokine receptor type 4 (CCR4) as an immunomodulatory target of hydroxychloroquine. The crystal structure of CCR4 was selected for molecular docking studies using the SwissDock modeling software. *In silico*, hydroxychloroquine interacts with Thr-189 within the CCR4 active site, presumably blocking endogenous ligand binding. However, the CCR4 antagonists compound 18a and K777 outperformed hydroxychloroquine *in silico*, demonstrating energetically favorable binding characteristics. Hydroxychloroquine may subject COVID-19 patients to QT-prolongation, increasing the risk of sudden cardiac death. The FDA-approved CCR4 antagonist mogalizumab is not known to increase the risk of QT prolongation and may serve as a viable alternative to hydroxychloroquine. Results from this report introduce additional FDA-approved drugs that warrant investigation for therapeutic use in the treatment of COVID-19.

## Introduction

The novel coronavirus SARS-CoV-2 (SARS-CoV-2; COVID-19) has emerged as an international outbreak of acute respiratory illness. The first case of COVID-19 was reported to the World Health Organization on December 31, 2019, although news reports have described the outbreak initiating as early as November 17, 2019 (Ma, 2020; Scher, 2020; Walker, 2020). While a majority of COVID-19 infections present as a mild disease, at risk patients may experience life-threatening symptoms, including fever, coughing, breathing difficulties, fatigue, myalgia, multi-organ failure, and sepsis. As of July 19, there has been a total of 3,698,161 confirmed cases of COVID-19 in the United States, with 139,659 reported deaths (Centers for Disease Control and Prevention, 2020). As a result, there are currently 242 clinical trials of COVID-19 treatments or vaccines that are either ongoing or recruiting patients (Live Science, 2020; RECOVERY, 2020). One treatment that has garnered international attention is hydroxychloroquine, a potent immunomodulatory agent FDA-approved for the treatment of malaria, lupus, and rheumatoid arthritis. Hydroxychloroquine has demonstrated promise *in vitro* and is currently being investigated in clinical trials for use as pre-exposure or post-exposure prophylaxis of COVID-19 infection, as well as in the treatment of patients with an active COVID-19 infection (World Health Organization, 2020). Results from a clinical trial hosted at Renmin Hospital of Wuhan University indicated that hydroxychloroquine treatment led to a reduction in time to clinical recovery (TTCR); reduced the body temperature recovery time, and shortened the duration of pneumonia relative to control subjects. In a small study commissioned by the French government, all patients taking the combination therapy hydroxychloroquine/azithromycin were virologically cured within 6 days of treatment (Gautret et al., 2020). Despite promising data in Europe and China, ongoing clinical trials in the United States have not yet demonstrated that hydroxychloroquine is safe and effective in the treatment of patients with COVID-19 (Food and Drug Administration, 2020).

Hydroxychloroquine was identified as a potential candidate for COVID-19 treatment due to its ability to efficiently interfere with the replication cycle of various parasites, fungi, bacteria, and viruses (Raoult et al., 1990; Raoult et al., 1999; Boulos et al., 2004; Rolain et al., 2007; Devaux et al., 2020). Preliminary data suggests that hydroxychloroquine related compounds impede COVID-19 infection in multiple ways: by increasing the endosomal pH required for virus-cell fusion and by interfering with the glycosylation of cellular receptors involved in SARS-CoV-2 spike protein cleavage-induced cell membrane fusion (Simmons et al., 2011; Chen et al., 2020; Meng et al., 2020; Wang et al., 2020). Additionally, chloroquine and hydroxychloroquine interfere with the proteolytic processing of M-protein and modify virion assembly and budding (Vincent et al., 2005; Wang et al., 2020). Notwithstanding promising data suggesting altered viral uptake, replication, and processing, the greatest benefit of hydroxychloroquine treatment may due to its potent immunomodulatory effects, given a majority of patients with severe COVID-19 infections succumb to cytokine release syndrome as a result of septic shock (Rainsfor et al., 2015; Liang et al., 2018; Ankrum, 2020; Moore and June, 2020). In addition to inhibiting normal lysosomal activity, it has been hypothesized that hydroxychloroquine modulates signaling pathways and the transcriptional regulation of genes responsible for governing cytokine production and regulating the activity of co-stimulatory effectors (Schrezenmeier and Dorner, 2020). However, despite being used in the treatment of numerous inflammatory and autoimmune conditions, the precise mechanism of action responsible for hydroxychloroquine induced immunomodulation remains unknown. In this study, we used an unbiased chemical similarity ensemble approach (SEA) to identify potential immunomodulatory targets of hydroxychloroquine.

## Materials and Methods

**Figure 1:**
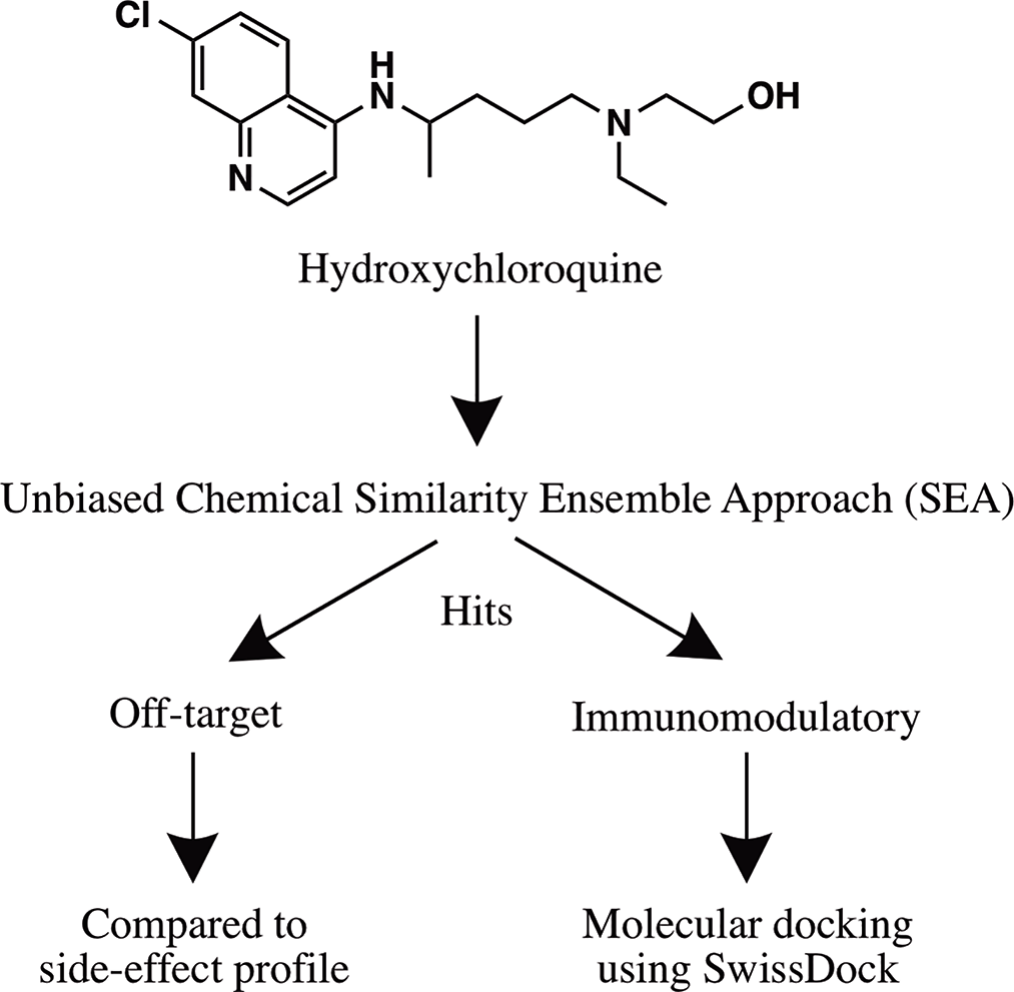
Flowchart summarizing the *in silico* approach employed to identify the immunomodulatory targets of hydroxychloroquine.

A step-wise *in silico* analysis was performed in order to uncover novel immunomodulatory targets of hydroxychloroquine (**Figure 1**).

### Similarity Ensemble Approach

The average FDA-approved drug has eight targets, making *in silico* target prediction of compounds an integral component of the drug discovery process (Ma’ayan et al., 2008; Santos et al., 2016; Wang et al., 2016). The chemical similarity ensemble approach (SEA) relates protein targets based on the set-wise chemical similarity among their ligands (Keiser et al., 2007). Using the ZINC database of over 2.7 million compounds, SEA is capable of generating highly accurate and robust predictions useful in drug repurposing and mechanism-of-action target prediction. SEA utilizes an unbiased approach; a single uniform submission query is employed to generate a list of prospective drug targets. The SMILES code of hydroxychloroquine [CCN(CCCC(C)NC1=C2C=CC(=CC2=NC=C1)Cl)CCO] (**Figure 1**) was submitted to the SEA server for target prediction. Predicted targets with maximum Tanimoto coefficient (MaxTc) values and p-values were generated. The MaxTc is defined as the percent chemical similarity of the investigational ligand relative to the most similar ligand for the target of interest, with scores ranging from 0 to +1 (where +1 is the highest similarity). A p-value of less than 1.000e-15 was used as the cut-off for statistical significance as previously described (Keiser et al., 2007; Wang et al., 2016).

### Molecular Docking

Molecular docking was performed using the SwissDock modeling software; a server dedicated to carrying out protein-ligand docking simulations hosted by the Swiss Bioinformatics Institute (Grosdidier et al., 2011; Bitencourt-Ferreira and Azevedo, 2019). The crystal structure of the chemokine receptor type 4 (CCR4) was selected for molecular docking assays (UniProt, 2020). Hydroxychloroquine was converted into a Mol.2 file using Marvin Sketch and submitted for molecular docking. Binding modes are scored using their FullFitness and clustered. Clusters are then ranked according to the average FullFitness of their elements with their respective Gibbs free energy (ΔG) values (Grosdidier et al., 2007). The ViewDock plug-in of UCSF Chimera was used to visualize predicted binding modes (Pettersen et al., 2004). Hydrogens were added in the ViewDock plug-in to optimize the predicted hydrogen bonding network and to allow UCSF Chimera to determine the protonation state (Lang, 2018). The known CCR4 antagonists AF-399, compound 18a, and K777 (**Figure S1**) were used as a positive control for comparison.

## Results

### Similarity Ensemble Approach

Results using the chemical Similarity Ensemble Approach (SEA) indicated six potential protein targets for hydroxychloroquine (**Table 1**). Two of the hits, including the alpha-1D adrenergic receptor (p = 0.004353) and muscarinic acetylcholine receptor M2 (p = 0.1283), did not possess significant p-values but have been verified *in vitro*. Four hits with significant p-values were discovered: Histidine-rich protein PFHRP-II (p = 1.63E-143), histamine N-methyltransferase (p=3.96E-53), DNA gyrase subunit B (3.08E-37), and C-C chemokine receptor type 4 (CCR4) (p = 3.79E-27). An additional 15 non-significant interactions were detected using SEA. Of the four hits with significant p-values, the target CCR4 presents as a target of interest due to its immunomodulatory function. CCR4 was selected for molecular docking assays using SwissDock and UCSF Chimera.

**Table 1:** Similarity Ensemble Approach Data for Hydroxychloroquine.

Target Name	Description	*P*	MaxTC
ADRA1D	Alpha-1D adrenergic receptor	0.004353	1.00
CHRM2	Muscarinic acetylcholine receptor M2	0.1283	1.00
HPR1	Histidine-rich protein PFHRP-II	1.63E-143	0.86
HNMT	Histamine N-methyltransferase	3.96E-53	0.51
gyrB	DNA gyrase subunit B	3.08E-37	0.29
CCR4	C-C chemokine receptor type 4	3.79E-27	0.47
CACNA2D1	Voltage-dependent calcium channel subunit alpha-2/delta-1	4.11E-15	0.45
NTSR1	Neurotensin receptor type 1	1.90E-11	0.32
AMP1	M1 family aminopeptidase	2.36E-09	0.39
APH1A	Gamma-secretase subunit APH-1A	1.16E-07	0.32
APH1B	Gamma-secretase subunit APH-1B	1.16E-07	0.32
NCSTN	Nicastrin	1.16E-07	0.32
PSEN2	Presenilin-2	1.16E-07	0.32
PSENEN	Gamma-secretase subunit PEN-2	1.17E-07	0.32
PSEN1	Presenilin-1	2.16E-07	0.32
PRNP	Major prion protein	2.24E-07	0.86
CDK11A	Cyclin-dependent kinase 11A	1.02E-06	0.30
CDK13	Cyclin-dependent kinase 13	1.02E-06	0.30
Abcc8	ATP-binding cassette sub-family C member 8	1.78E-06	0.29
CDK11B	Cyclin-dependent kinase 11B	1.95E-06	0.30
ANPEP	Aminopeptidase N	4.12E-06	0.39

Results highlighted in blue indicated verified hits in vitro. Targets highlighted in green are considered to be statistically significant (p < 1.000E-15). Results in white indicate predicted targets that are not considered to be statistically significant by conventional criteria.

### Molecular Docking

**Figure 2:**
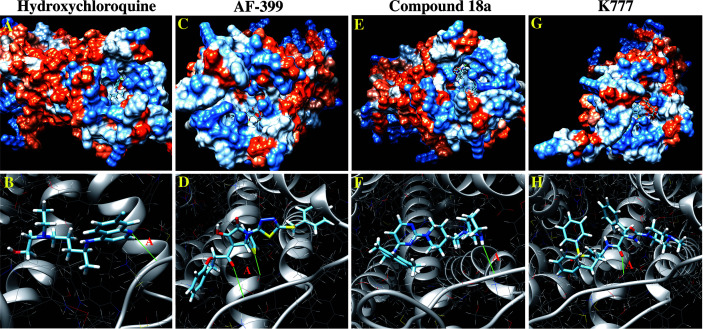
*In Silico* docking images for hydroxychloroquine and controls against CCR4. **(A)** Hydrophobicity plot demonstrating hydroxychloroquine interacting with the CCR4 active site. **(B)** Hydroxychloroquine forms a hydrogen bond with Thr-189 (A) in the CCR4 active site, as depicted in green. **(C)** Hydrophobicity plots demonstrating AF-399 interacting with the CCR4 active site. **(D)** AF-399 forms a hydrogen bond with Thr-189 (A) in the CCR4 active site. **(E)** Hydrophobicity plots demonstrating compound 18a interacting with the CCR4 active site. **(F)** Compound 18a forms a hydrogen bond with Thr-189 (A) in the CCR4 active site. **(G)** Hydrophobicity plots demonstrating K777 interacting with the CCR4 active site. **(H)** K777 forms a hydrogen bond with Thr-189 (A) in the CCR4 active site.

A total of 42, 35, 41, and 32 binding clusters were calculated for hydroxychloroquine, AF-399, compound 18a, K777, respectively. Clusters were then ranked according to the average FullFitness of their elements beginning with cluster 0/element 0 and their corresponding Gibbs free energy (ΔG) values. More negative ΔG values indicate favorable binding characteristics. At cluster 0/element 0, hydroxychloroquine demonstrated a ∆G of -7.91, whereas positive controls AF-399, compound 18a, and K777 had ∆G values of -8.39, -8.93, and -8.75, respectively (**Table 2**). The average ∆G of hydroxychloroquine was -7.06, whereas AF-399, compound 18a, and K777 had average ∆G values of -7.93, -7.81, and -8.47, respectively. UCSF Chimera was used to visualize the predicted binding conformations of hydroxychloroquine and controls. All drugs interact with the active site of CCR4 (**Figure 2**). All compounds formed a hydrogen bond with Thr-189. In cluster 3/element 6, hydroxychloroquine also interacted with Lys-35. At cluster 1/element 0, K777 interacted with both Thr-189 and Cys-110. Lastly, in clusters 4 and 5, compound 18a interacted with Thr-189, Pro-171, and Cys-187.

**Table 2:** Molecular Docking Data Summary for Hydroxychloroquine and Plerixaflor Against CXCR4.

	Cluster 0/Element 0			Average ΔG Value	
Compound	CCR4 IC_50_	FullFitness (kcal/mol)	ΔG (kcal/mol)	FullFitness (kcal/mol)	ΔG (kcal/mol)
Hydroxychloroquine	N/A	-1415.24	-7.91	-1407.70	-7.06
AF-399	2-10 nM	-1441.47	-8.39	-1432.30	-7.93
Compound 18a	8.1 nM	-1497.75	-8.93	-1485.33	-7.81
K777	N/A	-1409.69	-8.75	-1401.83	-8.47

In vitro and in silico data for hydroxychloroquine and controls. The CCR4 IC50 value of hydroxychloroquine and K777 is unknown, whereas AF-399 and compound 18a demonstrate exceptional IC50 in the low nanomolar range. The FullFitness score summarizes the total number of ligand-to-protein interactions. A more favorable binding mode is indicated by a more negative FullFitness score. Cluster 0/Element 0 indicates the docking score that corresponds to the peak FullFitness score for each respective compound. All compounds demonstrate similar FullFitness scores in cluster 0/element 0, whereas controls compound 18a and K777 demonstrated exceptional Gibb’s Free Energy (ΔG) values. Similar to the FullFitness score, a more negative ΔG value indicates an energetically favorable binding interaction. Based on ΔG values, K777 demonstrated the most impressive docking scores in silico.

## Discussion

Results from the present study indicate that the FDA-approved drug hydroxychloroquine interacts with the C-C chemokine receptor type 4 (CCR4) *in silico*. CCR4 is a seven transmembrane G protein-coupled receptor (GPCR) expressed throughout the human body with highest expression levels in the bone marrow and lymphoid tissue (CCR4 n.d.). Its endogenous ligands, the chemokines CCL17 and CCL22, are regulators of the immune system, contributing to lymphocyte trafficking and cytokine release. CCR4 plays both beneficial and deleterious effects in the immune response. Furthermore, CCR4 plays a beneficial role in recruiting immune cells to the site of infection, triggering a downstream immune response; however, CCR4 can also play deleterious roles, negatively impacting wound healing and contributing to sepsis (Barros et al., 2019; Traeger et al., 2008). Additionally, CCR4 induces Th2 effector T-cell polarization, suppressing Th1-dependent cellular immune responses that protect the body against pathogenic substances (Bayry et al., 2008; Szabo et al., 2003). In rheumatoid arthritis, CCR4 plays a critical role in CD4+ T cell migration to the synovium contributing to joint inflammation (Yang et al., 2004). In patients with rheumatoid arthritis and lupus, the percentage of circulating CD4+/CCR4+ T cells is significantly elevated relative to heathy controls (Yang et al., 2004). For patients with lupus in particular, an increase in CCR4-expressing Th2 cells are increased in patients with active disease and is thought to play a role in the development of autoantibodies contributing to disease pathogenesis (Hase et al., 2001). Additionally, significant numbers of CD8+/CCR4+ T lymphocytes were identified in skin biopsies of patients with cutaneous lupus erythematosus (CLE) and correlated with aggressive skin scarring (Wenzel et al., 2005). The putative ability of hydroxychloroquine to inhibit CCR4 signal transduction likely explains its efficacy in the treatment of rheumatoid arthritis and lupus. A recent study investigating the immune cell transcriptome profiles of COVID-19 patients with pneumonia identified CCR4 expression as an upregulated target relative to healthy controls (De Basi et al., 2020). CCR4 receptor antagonists have demonstrated efficacy in the prevention and treatment of septic shock (Power et al., 2000). In a lipopolysaccharide model of sepsis, transgenic CCR4-/- knockout mice showed improved survival rates due to a significant reduction in pro-inflammatory cytokine release and associated sepsis (Traeger et al., 2008). These data suggest a potential role for the use of CCR4 antagonists in the treatment of COVID-19 patients.

The chemical similarity ensemble approach (SEA) yielded interactions with several off-target proteins that likely contribute to the side-effect profile of hydroxychloroquine. Two of the hits, including the alpha-1D adrenergic receptor and muscarinic acetylcholine receptor M2, have been verified *in vitro* (Auerbach, 2020; Gordon et al., 2020). Inhibition of the alpha-1D adrenergic receptor may explain the rare, but reported, side-effects such as palpitations, headaches, and muscle weakness [Drugs.com, 2020j (Hydroxychloroquine)]. Side-effects such as blurred vision, constipation, dry mouth, and urinary retention are likely a result of the anti-cholinergic manifestations associated with muscarinic M2 receptor interaction. Also noteworthy, inhibition of several cyclin dependent kinases (CDKs), such as CDK11A, CDK11B, and CDK13 may explain the commonly reported gastrointestinal side-effects such as diarrhea, nausea, and vomiting.

Based on our molecular docking results, the control CCR4 inhibitors AF-399, compound 18a, and K777 demonstrate more pronounced thermodynamic binding characteristics to CCR4 relative to hydroxychloroquine. CCR4 antagonists are used in the United States for the treatment of mycosis fungoides and Sézary disease, and used globally for the treatment of adult T-cell leukemia/lymphoma (ATCLL) and CCR4+ cutaneous T cell lymphoma (CTCL) (Yu et al., 2007; FDA, 2018). AF-399 and compound 18a exhibit exceptional binding characteristics against CCR4 in vitro (IC50 = 2-10 and 8.1 nM, respectively) (Bayry et al., 2008; Klein et al., 2017; Shukla et al., 2016). Additionally, K777 is a broad-spectrum antiviral that prevents cathepsin-mediated cell entry (Jacobsen et al., 2000). K777 inhibits pseudovirus entry of SARS-CoV and EBOV with IC50 values of 0.68 nM and 0.87 nM, respectively (Jacobsen et al., 2000; Sato et al., 2013; Zhao, et al., 2015). Unlike hydroxychloroquine, none of these compounds, nor the FDA-approved CCR4 antagonist mogamulizumab, are known to increase the risk of QT prolongation (Mazzanti et al., 2020). Excessive QT prolongation can trigger life threatening arrhythmias such as torsades de pointes. It is important to note that hydroxychloroquine has been used for over half a century and has a well-established safety profile in the use for chronic conditions such as rheumatoid arthritis and lupus. Principally relevant to the current pandemic, a majority of patients hospitalized for COVID-19 infection have underlying comorbidities, such as cardiovascular disease, and are more likely to be elderly. Thus, confounding variables exist that may increase patient risk of adverse effects following hydroxychloroquine administration in the acute management of COVID-19 infection. Drugs that inhibit CCR4 in absence of QT prolongation, such as mogalizumab, may serve as viable alternatives to hydroxychloroquine in the treatment of patients with severe COVID-19 manifestations.

In addition to CCR4 inhibitors, the evaluation of various other classes of anti-inflammatory agents, particularly those drugs that limit cytokine release, may show promise in the treatment of COVID-19 infections. There are currently 242 clinical trials of COVID-19 treatments or vaccines (RECOVERY, 2020). Notable drugs include hydroxychloroquine, dexamethasone, and remdesivir (**Table S1**) (Stewart, 2020). Particularly noteworthy, the use of dexamethasone reduced death by up to one third in hospitalized patients with severe respiratory complications of COVID-19. A study in Wuhan, China demonstrated that plasma concentrations of interleukin (IL)-2, IL-7, IL-10, granulocyte-colony stimulating factor (GCSF), interferon gamma-induced protein 10 (IP-10 or CXCL10), monocyte chemoattractant protein-1 (MCP1), macrophage inflammatory protein 1-alpha (M1P1A), and tumor necrosis factor-alpha (TNF-α) were statistically elevated in ICU patients relative to non-ICU patients (Huang et al., 2020). While IL-2, CXCL10, and TNF-α contribute to the pathogenesis of sepsis, IL-7, IL-10, GCSF, MCP1, M1P1A are likely elevated as an endogenous attempt to combat sepsis (Snydman et al., 1990; Latifi et al., 2002; Takahashi et al., 2002; Schefold, 2011; Gaballa et al., 2012; Gomes et al., 2013; Herzig et al., 2014; Francois et al., 2018). IL-2 promotes the proliferation of T and B lymphocytes (Ross and Cantrell, 2018). While IL-2 plays a beneficial role in preventing autoimmune disease development, high levels of IL-2 lead to capillary leak syndrome and are associated with nosocomial sepsis (Snydman et al., 1990). FDA-approved IL-2 inhibitors that may warrant investigation in the treatment of patients with severe coronavirus disease include basiliximab and daclizumab [Drugs.com, 2020d (basiliximab); Drugs.com, 2020g (Daclizumab)] (**Table S2**). The chemokine CXCL10 (IP-10) plays a deleterious role in infection and inflammation by activating the chemokine receptor CXCR3, an important regulator of lymphocyte trafficking and activation (Herzig et al., 2014). The experimental compound ganodermycin is a potent inhibitor of the chemoattractant CXCL10 and may benefit patients with severe COVID-19 infection (Jung et al., 2011). Additionally, the FDA-approved 3-hydroxy-3-methylglutaryl-CoA (HMG-CoA) reductase inhibitor atorvastatin inhibits CXCL10 and may be useful in the treatment of COVID-19 (Grip and Janciauskiene, 2009). Tumor necrosis factor alpha (TNF-α) is an endogenous pyrogen which mediates fever and inflammatory processes (Stefferl et al., 1996; Luheshi et al., 1997). TNF-α inhibitors have also proven beneficial in treating cytokine storm, a common cause of death in patients with severe coronavirus disease (Gaballa et al., 2012). TNF-α inhibitors, such as adalimumab, certolizumab, etanercept, golimumab, and infliximab may serve as prospective therapeutics in COVID-19 treatment [Drugs.com, 2020a (Adalimumab); Drugs.com, 2020f (Certolizumab); Drugs.com, 2020h (Etanercept); Drugs.com, 2020i (Golimumab); Drugs.com, 2020k (Infliximab)]. Additionally, inhibitors of pro-inflammatory targets such as interleukin (IL)-1 beta (IL-1β), IL-6, and Janus Kinase (JAK) 1/2 may be useful in reducing fever and preventing cytokine storm (Bird, 2018; Dinarello, 2015; Ekilsson et al., 2014; Gabay et al., 2010; Roskoski, 2016; Drugs.com, 2020b; Drugs.com, 2020c; Drugs.com, 2020e; Drugs.com, 2020l; Drugs.com, 2020m; Drugs.com, 2020n; Drugs.com, 2020o). (**Table S2**).

In summary, the use of hydroxychloroquine in the treatment of COVID-19 has received significant media attention over the past few months. Despite promising data from clinical trials hosted in China and France, hydroxychloroquine has not yet proven to be safe in preliminary U.S. clinical trials. QT prolongation in patients taking hydroxychloroquine along with azithromycin, a combination that has proven effective in European clinical trials, has raised concern regarding the use of these medicines in COVID-19 treatment (Mazzanti et al., 2020). This study has identified CCR4 as the immunomodulatory target for hydroxychloroquine. CCR4 antagonists that do not promote QT prolongation, such as the FDA-approved drug mogamulizumab or experimental compound K777, may warrant investigation in the treatment of severe coronavirus disease as either monotherapy or in combination with antivirals. The administration of anti-inflammatory agents may improve health outcomes in patients at risk of cytokine storm and warrant further investigation in clinical trials.

Limitations of the present study include a lack of *in vitro* verification of CCR4/hydroxychloroquine interaction. The use of the unbiased similarity ensemble approach (SEA) demonstrated that CCR4 is an immunomodulatory target for hydroxychloroquine. Results from this study may help identify FDA-approved drugs suitable for repurposing in the treatment of severe COVID-19 infection.

## Data Availability Statement

The datasets presented in this study can be found in online repositories. The names of the repository/repositories and accession number(s) can be found in the article/**Supplementary Material**.

## Author Contributions

All authors listed have made substantial, direct and intellectual contribution to the work and approved it for publication. TB and RN arranged the research, designed the experiments, examined and construed the data, wrote the manuscript, provided financial support, and guided all the experiments. KB, CH, RH, and TD examined and construed the data, and wrote and edited the manuscript. 

## Funding

This work was supported by an NIH training grant HL007260 to TB. The funders had no role in study design, data collection and analysis, decision to publish, or preparation of the manuscript.

## Conflict of Interest

The authors declare that the research was conducted in the absence of any commercial or financial relationships that could be construed as a potential conflict of interest.
