# Supplementary material for: The C-C Chemokine Receptor Type 4 Is an Immunomodulatory Target of Hydroxychloroquine
Source: Front Pharmacol. 2020 Aug 28;11:1253. doi: 10.3389/fphar.2020.01253 (PMC7482581; doi:10.3389/fphar.2020.01253)
Supplement: Supplementary file 1 [file DataSheet_1.pdf]

**Table S1. Summary of noteworthy drugs currently under investigation in clinical trials for the treatment of COVID-19 and related complications.**

| Generic name | Brand name | Therapeutic Class | Mechanism of action | Labeled Indication(s) |
|---|---|---|---|---|
| Hydroxychloroquine[1] | Plaquenil[2] | Antimalarial agent; aminoquinoline | Increases pH of digestive vacuoles within sensitive malarial parasites and interferes with lysosomal degradation of hemoglobin. Mechanisms underlying the anti-inflammatory and immunomodulatory effects are unknown. | Malaria, lupus erythematosus, rheumatoid arthritis |
| Azithromycin[3] | Zithromax; Zmax | Macrolide antibiotic | Binds to the 50S ribosomal subunit resulting in blockage of transpeptidation. | Chancroid, chronic obstructive pulmonary disease, acute exacerbation, *Mycobacterium avium* complex, otitis media, acute, community-acquired pneumonia, skin and skin structure infection, uncomplicated Streptococcal pharyngitis (group A), urethritis/cervicitis |
| Remdesivir[4] | | Antiviral agent | Not fully elucidated; inhibits RNA synthesis. | |
| Dexamethasone[5] | Decadron | Corticosteroid | Immunosuppression; suppresses migration of neutrophils and decreases lymphocyte colony proliferation | Inflammatory skin and allergy conditions; ulcerative colitis, arthritis, lupus, psoriasis, and breathing disorders. |

[1]Hydroxychloroquine. Lexi-Drugs. Lexicomp. Wolters Kluwer. Hudson, Oh. Available at https://online.lexi.com. Accessed April 25, 2020.

[2]Plaquenil (hydroxychloroquine) [package insert]. Princeton, NJ; Dr. Reddy's Laboratories Inc.; Revised July, 2019.

[3]Azithromycin. Lexi-Drugs. Lexicomp. Wolters Kluwer. Hudson, Oh. Available at https://online.lexi.com. Accessed April 25, 2020.

[4]Remdesivir. Lexi-Drugs. Lexicomp. Wolters Kluwer. Hudson, Oh. Available at https://online.lexi.com. Accessed April 25, 2020.

[5]Dexamethasone. Lexi-Drugs. Drugs.com. Available at https://www.drugs.com/dexamethasone.html. Accessed July 22, 2020.
