# Supplementary material for: The C-C Chemokine Receptor Type 4 Is an Immunomodulatory Target of Hydroxychloroquine
Source: Front Pharmacol. 2020 Aug 28;11:1253. doi: 10.3389/fphar.2020.01253 (PMC7482581; doi:10.3389/fphar.2020.01253)
Supplement: Supplementary file 2 [file DataSheet_2.pdf]

Supplemental Table 2. FDA-approved immunomodulatory drugs that may serve useful in the treatment of severe COVID-19 infection.

Generic name	Brand name	Mechanism of action	Labeled Indication(s)
**Basiliximab**	**Simulect**	**IL-2 inhibitor**	**Organ transplantation.**
**Daclizumab**	**Zinbryta**	**IL-2 inhibitor**	**Multiple sclerosis, relapsing.**
**Ganodermycin**	**N/A**	**CXCL10 inhibitor**	**N/A**
**Atorvastatin**	**Lipitor**	**HMG-CoA reductase inhibitor and CXCL10 inhibitor**	**High cholesterol and to lower the risk of stroke, heart attack, or other heart complications in people with type 2 diabetes, coronary heart disease, or other risk factors.**
**Adalimumab**	**Humira**	**TNF-α inhibitor**	**Rheumatoid arthritis, psoriatic arthritis, ankylosing spondylitis, Crohn's disease, ulcerative colitis, psoriasis, hidradenitis suppurativa, uveitis, and juvenile idiopathic arthritis.**
**Certolizumab**	**Cimzia**	**TNF-α inhibitor**	**Crohn's disease, rheumatoid arthritis, psoriatic arthritis, ankylosing spondylitis, and plaque psoriasis.**
**Etanercept**	**Enbrel**	**TNF-α inhibitor**	**Rheumatoid arthritis, plaque psoriasis, and psoriatic arthritis.**
**Golimumab**	**Simponi**	**TNF-α inhibitor**	**Rheumatoid arthritis and ankylosing spondylitis.**
**Infliximab**	**Xeljanz**	**TNF-α inhibitor**	**Rheumatoid arthritis, psoriatic arthritis, ankylosing spondylitis, plaque psoriasis, Crohn's disease and ulcerative colitis.**
**Rilonacept**	**Arcalyst**	**IL-1β inhibitor**	**Familial Cold Auto-inflammatory Syndrome (FCAS) and Muckle-Wells Syndrome (MWS).**
**Canakinumab**	**Ilaris**	**IL-1β inhibitor**	**Familial Cold Auto-inflammatory Syndrome (FCAS), Muckle-Wells Syndrome (MWS), Tumor Necrosis Factor Receptor Associated Periodic Syndrome (TRAPS), Hyperimmunoglobulin D Syndrome (HIDS), and Familial Mediterranean Fever (FMF).**
**Anakinra**	**Kineret**	**IL-1R inhibitor**	**Rheumatoid arthritis and Neonatal-Onset Multisystem Inflammatory Disease (NOMID).**
**Tocilizumab**	**Actermra**	**IL-6 inhibitor**	**Rheumatoid arthritis and giant cell arteritis.**
**Siltuximab**	**Sylvant**	**IL-6 inhibitor**	**Multicentric Castleman's Disease (MCD).**
**Ruxolitinib**	**Jakafi and Jakavi**	**JAK1/JAK2 inhibitor**	**Myelofibrosis, polycythemia vera, and graft-versus-host disease.**
**Barcitinib**	**Olumiant**	**JAK1/JAK2 inhibitor**	**Rheumatoid arthritis.**

FDA-approved immunomodulatory drugs that may warrant further investigation include rilonacept, canakinumab, anakinra, tocilizumab, siltuximab, golimumab, infliximab, ruxolitinib, and baricitinib. Rilonacept and canakinumab are human monoclonal antibodies targeted at neutralizing interleukin-1 beta (IL-1β) [Drugs.com, 2020 (Rilonacept); Drugs.com, 2020 (Canakinumab)]. Anakinra interacts with the IL-1 receptor, blocking IL-1α and IL-1β from binding [Drugs.com, 2020 (Anakinra)]. IL-1 pathways are implicated in multiple inflammatory processes, known for triggering fever production and implicated in cytokine release syndrome in humans [Gabay et al., 2010; Dinarello, 2015; Bird, 2018]. Targeting IL-6 using drugs such as tocilizumab or siltuximab may also help to control inflammation and reduce fever in patients with COVID-19. Interleukin-6 (IL-6) is an important mediator of fever by initiating the synthesis of hypothalamic prostaglandin E2 (PGE2), thereby changing the body's temperature set point [Drugs.com, 2020 (Tocilizumab); Drugs.com, 2020 (Siltuximab)]. Lastly, Janus kinase 1 and 2 (JAK1/2) inhibition with drugs such as ruxolitinib and baricitinib may serve as a useful approach in controlling inflammation in patients with severe COVID-19 infection [Drugs.com, 2020 (Ruxolitinib); Drugs.com, 2020 (Barcitinib)]. JAK1/2 are critical effectors of pro-inflammatory cytokine signaling and regulate immune function [Roskoski, 2016; Kohler et al., 2017]. JAK1/2 inhibition may be useful in controlling cytokine release in patients at risk of cytokine storm.
