# Supplementary figures and images for: The C-C Chemokine Receptor Type 4 Is an Immunomodulatory Target of Hydroxychloroquine
Source: Front Pharmacol. 2020 Aug 28;11:1253. doi: 10.3389/fphar.2020.01253 (PMC7482581; doi:10.3389/fphar.2020.01253)

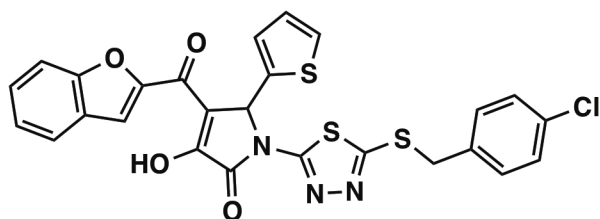

**AF-399**

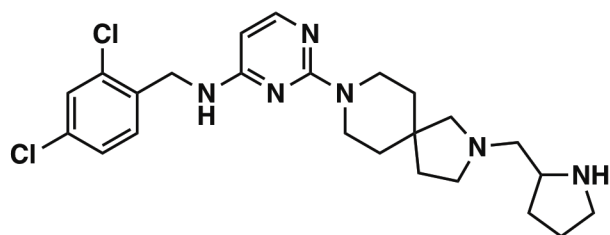

**Compound 18a**

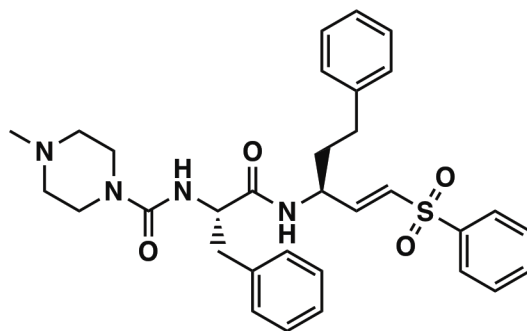

**K777**

**Figure S1.** Chemical Structures of CCR4 antagonists AF-399, compound 18a, and K777.

Supplement: Supplementary file 3 [file Image_1.pdf]
